# A Prospective, Randomized Trial of Bioresorbable Polymer Drug-Eluting Stents versus Fully Bioresorbable Scaffolds in Patients Undergoing Coronary Stenting

**DOI:** 10.3390/jcm13195949

**Published:** 2024-10-07

**Authors:** Jens Wiebe, Robert A. Byrne, Christian Bradaric, Constantin Kuna, Thorsten Kessler, Mathieu Pfleiderer, Sebastian Kufner, Erion Xhepa, Petra Hoppmann, Michael Joner, Heribert Schunkert, Karl-Ludwig Laugwitz, Adnan Kastrati, Salvatore Cassese

**Affiliations:** 1 Klinik für Herz- und Kreislauferkrankungen, Deutsches Herzzentrum München, Technische Universität München, Lazarettstrasse 36, 80636 Munich, Germany; jens.wiebe@googlemail.com (J.W.); kuna@dhm.mhn.de (C.K.); thorsten.kessler@tum.de (T.K.); mathieu.pfleiderer@gmail.com (M.P.); sebastian.kufner@tum.de (S.K.); erionxhepa@icloud.com (E.X.); michaeljoner@me.com (M.J.); schunkert@dhm.mhn.de (H.S.); kastrati@dhm.mhn.de (A.K.); 2 DZHK (German Centre for Cardiovascular Research), Partner Site Munich Heart Alliance, 81377 Munich, Germany; kl.laugwitz@mri.tum.de; 3 Cardiovascular Research Institute, Mater Private Hospital, D07 WKW8 Dublin, Ireland; robert.byrne@materprivate.ie; 4 School of Pharmacy and Biomolecular Sciences, RCSI University of Medicine and Health Sciences, D02 YN77 Dublin, Ireland; 5 1. med. Klinik, Klinikum Rechts der Isar, Technische Universität München, 81675 Munich, Germany; bradaric@tum.de (C.B.); petra.hoppmann@t-online.de (P.H.)

**Keywords:** drug-eluting stent, percutaneous coronary intervention, scaffold

## Abstract

**Background:** The performance of an everolimus-eluting bioresorbable scaffold (BRS) was inferior to an everolimus-eluting metallic drug-eluting stent (DES) with permanent polymer, mainly due the mechanical features of BRS technology. The performance of BRS as compared to metallic DES with bioresorbable polymers remains unstudied. **Methods:** This prospective, randomized, multicenter, clinical trial enrolled patients who underwent coronary stenting for de novo coronary lesions. Patients were randomly assigned to bioresorbable polymer everolimus-eluting stents (BP-EES) or everolimus-eluting BRS. The primary endpoint was percentage diameter stenosis (in-device) at 6- to 8-month angiographic surveillance. The main secondary endpoint was the device-oriented composite endpoint (DOCE) of cardiac death/target vessel-myocardial infarction/target lesion revascularization assessed after 12 months and 5 years. **Results:** The trial was prematurely terminated after the enrollment of 117 of 230 patients (BP-EES, n = 60; BRS, n = 57) due to safety issues associated with BRS technology. The primary endpoint of in-device diameter stenosis at angiographic surveillance was 12.5 ± 7.7% with BP-EES versus 19.3 ± 16.5% with BRS (*p* = 0.01). The DOCE occurred in 5.0% in the BP-EES group versus 12.3% of patients in the BRS group (hazard ratio [HR] 2.48, 95% confidence interval [CI] 0.64–9.58, *p* = 0.19) after 12 months and in 11.7% in the BP-EES group versus 26.4% of patients in the BRS group (HR 2.38, 95% CI 0.97–5.84, *p* = 0.06) after 5 years. **Conclusions:** BP-EES showed superior mid-term angiographic performance compared with BRS. Clinical event rates did not differ significantly between the groups up to 5 years of follow-up. These results should be interpreted with caution in view of the premature discontinuation of the study.

## 1. Introduction

Drug-eluting stent (DES) implantation represents the strategy of choice for patients undergoing percutaneous coronary intervention (PCI) [1]. However, effective neointimal suppression after DES occurs at the expense of delayed arterial healing [2]. This phenomenon underlies an increased risk of thrombotic events as well as a possible excess of in-stent neoatheroma formation over the long term [3,4]. To address this issue, technological advances have focused on DES with bioresorbable polymer coatings [5] and fully bioresorbable backbones [6].

DES with bioresorbable polymer coatings have shown to improve vascular healing after coronary stenting [5]. In particular, a thin-strut bioresorbable polymer everolimus-eluting stent (BP-EES) demonstrated improved angiographic and clinical performance [7,8]. Fully bioresorbable scaffolds were an alternative to metallic DESs to provide short-term scaffolding and drug delivery with potentially improved vascular healing. The everolimus-eluting poly-L-lactic-based bioresorbable scaffold (BRS) is the most extensively studied device in this class [6]. Despite initially promising results in selected patients, randomized controlled trials with broader inclusion criteria have shown higher event rates in patients treated with BRS than after stent implantation with metal DES during short- and long-term follow-up [9,10]. In particular, the thrombosis rate was consistently higher in the BRS groups, which is why the device is no longer commercially available.

As most randomized studies with BRS have used a permanent polymer DES as a comparator, the performance of BRS compared to metallic DES with bioresorbable polymers has yet to be investigated. This comparison could be of interest for the future development of technologies based on fully bioresorbable backbones [11,12]. In this regard, we present the results of the Intracoronary Stenting and Angiographic Results: Test Efficacy of Stents with Bioresorbable Polymer Coating Versus Bioresorbable Polymer Backbone (ISAR-RESORB) randomized trial, which compared the angiographic and clinical performance of BP-EES versus BRS for de novo coronary lesions in patients amenable to PCI.

## 2. Methods

### 2.1. Study Design

The ISAR-RESORB study is a multicenter, prospective, randomized, open-label, superiority, clinical trial of BP-EES versus BRS in patients undergoing PCI for de novo coronary lesions (NCT02421016). The study fully adheres to the principles outlined in the Declaration of Helsinki. Approval for the protocol and informed consent form was obtained from the local ethics committee. A further vote from the ethics committee was obtained for the 5-year follow-up, since this was not part of the initial protocol. All participants or their legally authorized representatives received and signed a written informed consent.

### 2.2. Participants and Interventions

Patients ≥18 years of age and with ischemic symptoms or evidence of myocardial ischemia in the presence of ≥50% de novo stenosis located in native coronary vessels with a reference diameter ≥2.5 mm and ≤3.9 mm and a lesion length <28 mm were eligible. The major exclusion criteria were the following: cardiogenic shock, acute ST-elevation myocardial infarction within ≤48 h from symptom onset, renal insufficiency (most recent serum creatinine within the last 72 h prior to randomization >2 mg/dL or 177 µmol/L), malignancies or other co-morbid conditions with life expectancy ≤12 months or that may result in protocol non-compliance, pregnancy (present, suspected or planned), breast feeding in women with childbearing potential, contraindications or allergies to any components of the study devices or the inability to take antiplatelet therapy for ≥6 months after stenting. Angiographic exclusion criteria comprised the following: target lesions located in the left main trunk or a bypass graft, severely calcified lesions, target lesions containing a side branch with a diameter ≥2 mm, ostial lesions, and severe vessel tortuosity.

Immediately after the decision to perform PCI, patients received 500 mg aspirin intravenously (if they did not receive it within the prior 12 h) and body weight-adjusted intra-arterial or intravenous heparin or bivalirudin. Glycoprotein IIb/IIIa inhibitors were administered at the discretion of the operators. PCI was performed according to institutional guidelines and standards using approved devices in addition to the investigational devices. The choice of balloon and device diameter, length, and applied inflating pressure was also left to the discretion of the operator, according to the compliance charts provided by the manufacturer for each device. The same randomly assigned stent had to be used in all lesions in those patients who require stenting in multiple lesions. Use of more than one stent per lesion was allowed. After receiving a pre-procedural loading dose, aspirin indefinitely and a P2Y12 inhibitor (clopidogrel, prasugrel, or ticagrelor) were prescribed for all patients at daily maintenance doses using standard local practice and for at least 6 months. Further cardioactive medications were prescribed according to discretion of the patient’s physician. ECG recordings were performed immediately after intervention and then daily until discharge. Cardiac markers (CK, CK-MB, Troponin, and high-sensitivity Troponin where available) were determined on the day after intervention and thereafter daily until discharge as well as for all suspected ischemic events. Relevant data were collected by the personnel of the Clinical Data Management Centre (ISARESEARCH Center, Munich, Germany) and entered into a computer database.

### 2.3. Outcomes and Definitions

The primary endpoint of the ISAR-RESORB trial was the percentage diameter stenosis (in-device) at 6- to 8-month invasive surveillance as assessed by quantitative coronary angiography (QCA) analysis. Secondary clinical endpoints were the device-oriented composite endpoint (DOCE) of cardiac death/target vessel-myocardial infarction (MI)/target lesion revascularization (TLR), the patient-oriented composite endpoint (POCE) of death/any MI/all revascularization, the composite of cardiovascular death or MI, and the incidence of stent/scaffold thrombosis (ST) according to ARC criteria [13]. Clinical follow-up was conducted after 1 and 12 months by telephone interviews or visits to the practice according to the protocol. Long-term follow-up up to 5 years was not included in the original study protocol and was performed retrospectively. The basis for these data was regular patient contacts conducted as part of the clinical routine. Thus, the analyses of the long-term data presented in this study should be interpreted as exploratory.

All deaths were adjudicated as cardiovascular unless a non-cardiovascular cause could be clearly provided. If autopsy was performed, the autopsy reports were solicited for determination of the cause of death. The definition of MI used in this trial was according to the Third Universal Definition of Myocardial Infarction [14].

### 2.4. Sample Size, Randomization, and Outcomes Assessment

The hypothesis of the ISAR-RESORB trial was that BP-EES are superior to BRS in terms of diameter stenosis at 6- to 8-month angiography after PCI. The alternate hypothesis was that there was no difference between both devices. Assuming a mean diameter stenosis of 15% for BP-EES and 20% for BRS and a standard deviation of 12% in both groups, the two-group *t*-test with a two-sided significance level of 0.05 would have required a number of 92 patients per group to test the superiority hypothesis. . To account for the incompleteness of the angiographic follow-up, 230 patients were planned to be enrolled. The sample size was calculated with nQuery Advisor (version 4.0, Statistical Solutions, GraphPad Software DBA Statistical Solutions, 225 Franklin St FL 26 Boston, MA, 02110-2853, USA) according to the method of O’Brien and Muller.

In each participating center, a 1:1 allocation to treatment was made by a computer-generated sequence provided by the electronic case report form system immediately after the decision to proceed with PCI. Patients who met all of the inclusion criteria and none of the exclusion criteria were randomized in the order that they qualified. Patients were considered enrolled in the study and eligible for the final intention to treat analysis at the time of randomization. Time zero was defined as the time of randomization. Both treatment groups were studied concurrently.

For the angiographic outcomes, angiograms were assessed off-line at the centralQCA core laboratory at the ISARESEARCH Center, Munich, Germany. Therefore, an automated edge-detection system (QAngio XA version 7.1, Medis Medical Imaging Systems, Leiden, The Netherlands) was used by experienced operators. For calibration, the contrast-filled non-tapered catheter tip was used. Measurements were performed on cineangiograms recorded after the intracoronary administration of nitroglycerine. Baseline QCA measurements were performed using the single worst-view projection for the target lesion, and the same view projection was used for the measurements after intervention. In the follow-up angiogram, the QCA measurements were performed using the single worst-view projection at that time point. In the follow-up angiogram, the in-segment area included the treated area and the 5 mm margins proximal and distal to the treated area. For the clinical outcomes, the independent Event Adjudication Committee (EAC) was responsible for adjudicating clinical events relevant to the primary and secondary endpoints. Members of the committee were blinded to treatment allocation where feasible. The 5-year follow-up was not subject to EAC evaluation.

### 2.5. Statistical Analysis

Categorical variables are presented as frequencies and proportions and were compared by using the chi-square test or Fisher’s exact test, as appropriate. Continuous data are shown as means with standard deviation or median with interquartile range and were compared by using Student’s *t*-test or the nonparametric Wilcoxon rank-sum test. For the survival analysis and further secondary clinical endpoints, the Kaplan–Meier methods were used. Cox proportional hazards models were used to calculate hazard ratios (HR) with 95% confidence intervals (CI) and *p*-values for treatment effects. Sensitivity analysis was conducted by imputing missing data for the primary endpoint using all of the baseline clinical and angiographic variables with multiple imputation by chained equations (mice package). All tests were two-sided and an alpha level of 0.05 was considered statistically significant. All analyses were conducted with R (version 3.5.0, R Core Team, R Foundation, Vienna, Austria).

## 3. Results

Between May 2015 and April 2017, a total of 117 patients were enrolled at 2 high-volume centers in Munich, Germany. The study was terminated before completing the enrollment of the planned numbers of patients due to safety warnings from the Food and Drug Administration concerning BRS technology. Of all included patients, 60 were treated with BP-EES and 57 with BRS. The CONSORT diagram showing the flow of participants through each stage of the trial is displayed in the Supplemental Materials.

Regarding the baseline characteristics, no relevant differences were found between either of the groups. Overall, 29 (24.8%) were female and at admission 37 (31.9%) patients presented stable angina and 43 (37.1%) were asymptomatic. An overview of the clinical characteristics is displayed in Table 1.

A total of 63 lesions were treated with BP-EES and 59 lesions with BRS. A summary of the angiographic and procedural results is displayed in Table 2. No differences were noted between both groups regarding the angiographic characteristics of the target lesions, including qualitative and quantitative assessment. Pre-dilatation was more often performed in the BRS group (85.7% versus 98.3%; *p* = 0.02) as well as post-dilatation (65.1% versus 88.1%, *p* < 0.01). The post-intervention QCA analysis showed a larger in-device percentage diameter stenosis in the BRS group (10.2 ± 4.5% versus 13.7 ± 6.0%; *p* < 0.001).

**Figure 1:**
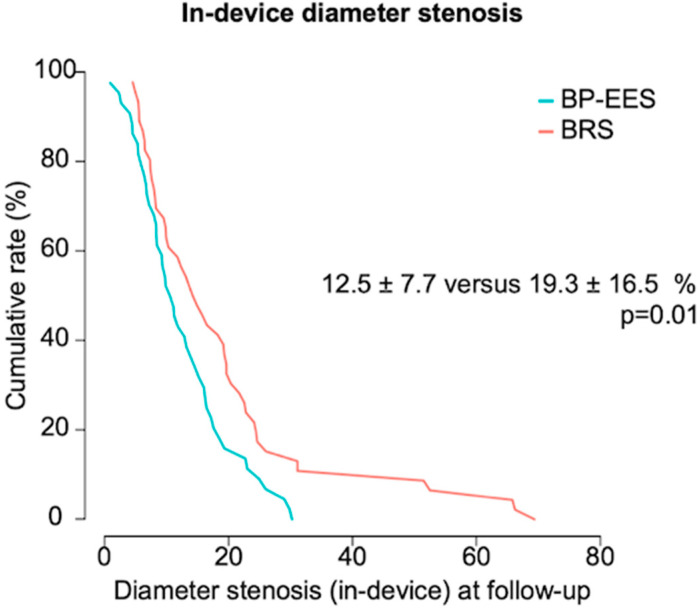
Primary endpoint of in-device percentage diameter stenosis. Cumulative frequency distribution curves for the primary endpoint of in-device percentage diameter stenosis at 6–8 month follow-up angiography. The *p*-value is derived from superiority testing.

An angiographic control was available in 44 lesions in the BP-EES group and in 46 lesions in the BRS group, with a median time to angiographic follow-up of 192 and 199 days, respectively. BP-EES were superior to BRS in terms of the primary endpoint of in-device percentage diameter stenosis (12.5 ± 7.7% versus 19.3 ± 16.5%; *p* = 0.01) (Figure 1). The rate of in-device binary restenosis was numerically lower in the BP-EES group (0% versus 10.9%; *p* = 0.07). In the in-segment analysis, no statistically relevant differences were found between both groups. An overview of the QCA analysis of the angiographic follow-up can be found in Table 3.

The 12-month clinical follow-up was available for all patients in both groups. Overall, no statistically relevant differences were found between either of the treatment arms. DOCE occurred in 3 patients (5.0%) in the BP-EES group and in 7 patients (12.3%) in the BRS group (HR 2.48, 95% CI 0.64–9.58, *p* = 0.19), whereas POCE occurred in 6 patients (10.0%) and in 10 patients (17.5%), in the BP-EES and BRS groups, respectively (HR 1.79, 95% CI 0.65–4.93, *p* = 0.26). No device thrombosis occurred among the treatment groups.

**Figure 2:**
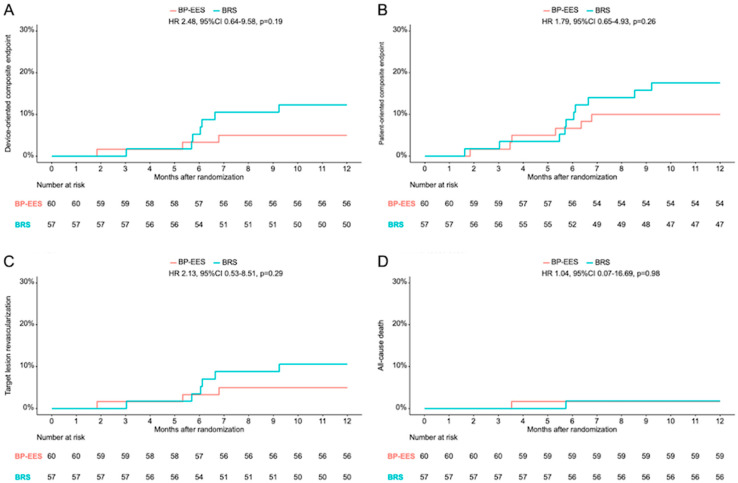
Kaplan–Meier time-to-event curves. Clinical outcomes at 12 months in patients treated with BP-EES versus BRS in terms of (**A**) the composite of cardiac death, target-vessel myocardial infarction, target lesion revascularization (device-oriented composite endpoint); (**B**) the composite of death, myocardial infarction, any revascularization (patient-oriented composite endpoint); (**C**) target lesion revascularization; and (**D**) all-cause death; data are shown as Kaplan–Meier event curves, with hazard ratios (95% confidence interval).

**Figure 3:**
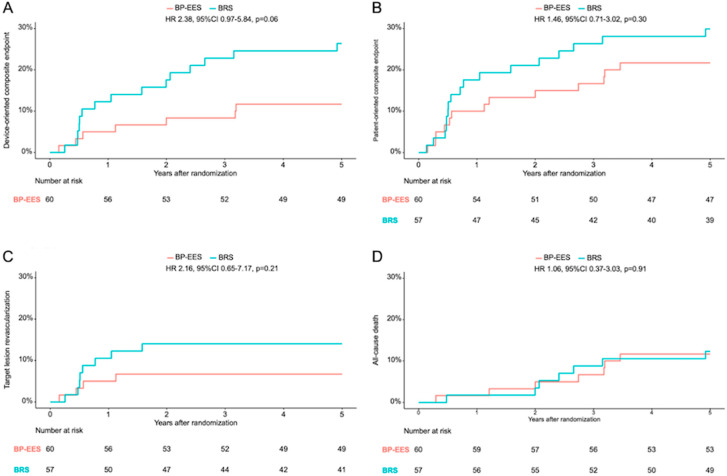
Kaplan–Meier time-to-event curves. Clinical outcomes at 5 years in patients treated with BP-EES versus BRS in terms of (**A**) the composite of cardiac death, target-vessel myocardial infarction, target lesion revascularization (device-oriented composite endpoint); (**B**) the composite of death, myocardial infarction, any revascularization (patient-oriented composite endpoint); (**C**) target lesion revascularization; and (**D**) all-cause death; data are shown as Kaplan–Meier event curves, with hazard ratios (95% confidence interval).

At 5-year follow-up, there were no statistically significant differences between groups. DOCE occurred in 7 patients (11.7%) in the BP-EES group and in 15 patients (26.4%) in the BRS group (HR 2.38, 95% CI 0.97–5.84, *p* = 0.06). POCE occurred in 13 patients (21.7%) in the BP-EES group and in 17 patients (29.9%) in the BRS group (HR 1.46, 95% CI 0.71–3.03, *p* = 0.30). No device thrombosis was observed in either group. Further clinical results are listed in Table 4 and time-to-event curves can be found in Figure 2 and Figure 3.

**Figure 4:**
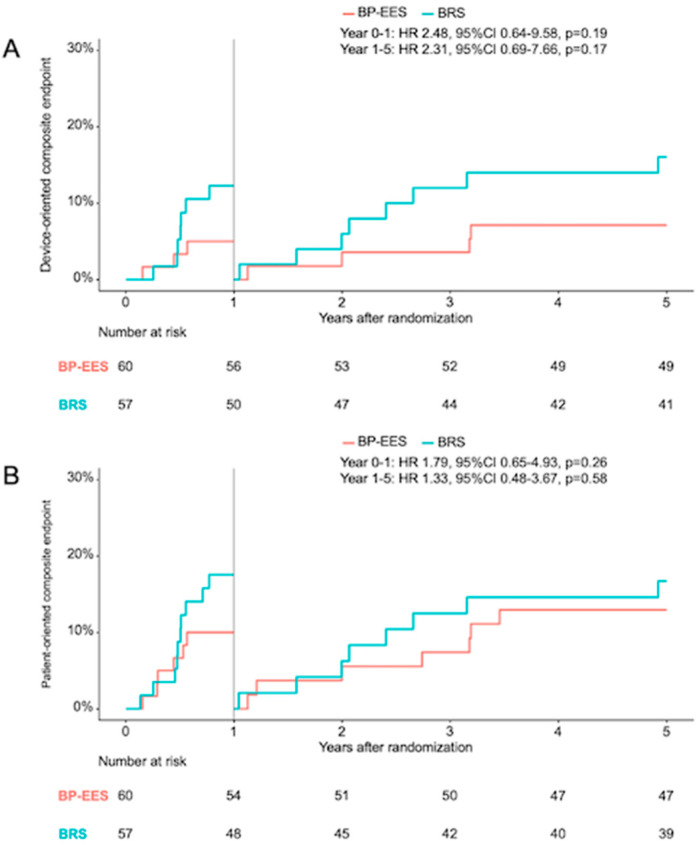
Landmark analysis. Landmark analysis of the device-oriented composite endpoint (**A**) and patient-oriented composite endpoint (**B**) shows the Kaplan–Meier event curves with hazard ratios (95% confidence interval) for the first year after the index procedure and from the one to five year clinical follow-up time frame.

The landmark analysis (Figure 4) for events between 1 and 5 years after the index procedure did not show any statistically relevant differences regarding DOCE (HR 2.31, 95% CI 0.69–7.66, *p* = 0.17), POCE (HR 1.33, 95% CI 0.48–3.67, *p* = 0.58), TLR (HR 2.24, 95% CI 0.20–24.74, *p* = 0.51) and all-cause death (HR 1.07, 95% CI 0.34–3.31, *p* = 0.91) between patients in the BP-EES group and BRS group.

## 4. Discussion

The main findings of the ISAR-RESORB study are the following:

(1) BP-EES have superior angiographic performance as compared to BRS, with a significantly lower in-device percentage diameter stenosis at 6- to 8-month invasive surveillance.
(2) Consistent with the angiographic data, the risk of DOCE after 12 months in the BRS group was doubled compared to BP-EES, although no statistically significant differences were found between either groups. This observation was also consistent throughout the 5-year clinical follow-up and the landmark analysis between 1 and 5 years.

The results of the ISAR-RESORB trial require careful discussion. The first study to report follow-up angiographic data on the BRS version studied in this trial was the ABSORB cohort B study, which included a total of 101 patients with non-complex coronary lesions. After 6 months, half of the participants underwent invasive surveillance which documented an in-device diameter stenosis of 19.2 ± 7.6%, comparable to that observed in the BRS group of the ISAR-RESORB study [15]. However, in the ISAR-RESORB study, the rate of in-device binary restenosis in the BRS group was 10.9%. The Comparison of Everolimus- and Biolimus-Eluting Coronary Stents with Everolimus-Eluting Bioresorbable Vascular Scaffold (EVERBIO II) randomized study included less selected patients, although most of the included lesions were not complex. At the 9-month angiographic follow-up, the diameter stenosis within the device was 16.9 ± 11.6% in the BRS group, significantly higher than in the comparator metallic DES platforms [16]. Overall, the angiographic results with BRS are in line and show consistently higher rates of in-device diameter stenosis out to approximately 6–9 months. However, these findings may potentially be explained by the thicker struts of the BRS (approximately 160 µm) compared to the standard metallic DES, which have usually a strut thickness of less than half of BRS [17].

Nevertheless, the angiographic superiority of DES did not result in superior clinical outcomes after 12 months in the ISAR-RESORB trial. The largest randomized study on patients treated with either BRS or DES is the randomized ABSORB III trial, which included a total of 2008 patients with stable coronary artery disease, of which 1322 patients were treated with BRS and 686 with DES. Similar to the ISAR-RESORB study, patients with bifurcation or ostial lesions, severe calcification, or presence of thrombus were excluded. After one year of clinical follow-up, no statistical difference was seen regarding the primary endpoint of target lesion failure (cardiac death, target vessel MI, TLR), though numerically higher event rates in the BRS group. Notably, the incidence of device thrombosis rate was twice as high in the BRS group as compared to a metallic everolimus-eluting stent [18]. A meta-analysis of 6 randomized trials and 3738 patients assessed the one-year clinical outcomes after implantation of BRS or metallic everolimus-eluting stent. Again, patients treated with BRS showed similar results in terms of target lesion failure, though a worryingly higher rate of device thrombosis was observed in patients treated with BRS [10]. In contrast with these observations, there was no evidence of BRS thrombosis in the ISAR-RESORB study, during the first year and throughout the observation period. The occurrence of BRS device thrombosis has been shown to be related to lesion selection and procedural factors, especially in the early phase after BRS implantation. A specific implantation protocol with careful pre- and post-dilation was able to mitigate the risk of BRS device thrombosis, especially within the first year [19,20], though the risk remained unchanged thereafter. In the ISAR-RESORB study, pre- and post-dilation were performed in nearly every patient assigned to BRS. Furthermore, lesions in the ISAR-RESORB study were highly selected: the reference diameter of 2.91 mm is larger than in other randomized studies [10] and BRS are thought to have an unfavorable outcome in small vessels. Finally, the ISAR-RESORB study excluded bifurcations and ostial lesions that are associated with a worse clinical outcome in the short term [21].

Notably, a large body of evidence exists on long-term follow-up data after BRS implantation. An individual-patient-data pooled meta-analysis of four randomized studies has revealed significantly higher rates for target lesion failure and device thrombosis 5 years after BRS therapy [22]. Above all, the rate of device thrombosis observed beyond 1 year was unexpected, as BRS was initially thought to reveal its vascular restorative advantages during this resorption period. It is assumed that the genesis of these late adverse effects is multifactorial. An optical coherence tomography study of 36 patients with late BRS device thrombosis identified BRS discontinuity as the most frequent cause of BRS device thrombosis, most likely due to an unfavorable and inhomogeneous resorption process. Less common reasons were malapposition and neoatherosclerosis [23]. Although no device thrombosis was found in the ISAR-RESORB study, higher overall event rates were also observed in the BRS group, and it must be assumed that the number of patients is too small to achieve statistical significance. These results are in keeping with the long-term follow-up data of the EVERBIO II trial, which found no acute coronary syndrome due to device thrombosis beyond 9 months [24].

### Limitations

When interpreting the results of the ISAR-RESORB study, some limitations must be taken into account. First, the study was stopped prematurely due to safety warnings from regulatory authorities regarding BRS technology. Furthermore, in April 2017, the BRS manufacturer advised customers to discontinue use of these devices immediately, in response to concerns about data showing high rates of thrombotic events with BRS compared to patients treated with metallic DES platforms, as discussed above [23]. This circumstance could be relevant for the analysis of the results. Secondly, due to the inclusion and exclusion criteria, several clinical and anatomical scenarios were not included in the present study. For example, patients with acute MI and ostial, bifurcation, long or calcified lesions were excluded. The subjects included in this study are highly selected and different from the patients observed in daily routine. Third, some patients did not receive a scheduled angiographic follow-up, although the proportion of patients with 6- to 8-month invasive surveillance in this study was close to 80% in both groups. Fourth, intravascular imaging was not mandatory, and the ISAR-RESORB trial can provide only a limited mechanistic perspective regarding device performance. Finally, the 5-year follow-up was not part of the original study protocol and was performed post hoc. In addition, the study was not designed to detect differences between the clinical outcomes of the two groups. For this reason, the clinical results should be interpreted with caution.

## 5. Conclusions

After enrolling half of the originally planned patients in this prospective, randomized trial including patients with predominantly non-complex coronary artery disease, BP-EES was superior to BRS with respect to the primary endpoint of percent diameter stenosis at a 6–8 months angiographic follow-up. However, this result did not lead to significantly higher event rates in the BRS group, and no device thrombosis occurred overall. Nevertheless, these results should be interpreted with caution given the early termination of the study due to safety concerns related to BRS. The role of technologies based on fully resorbable scaffolds with different design and resorption processes to BRS needs to be investigated in future randomized trials.

## Figures and Tables

**Table 1:** Baseline patient characteristics.

	BP-EES	BRS	*p* Value
Patients	60	57	
Age	66.7 ± 11.4	68.5 ± 10.5	0.38
Female gender	13 (21.7)	16 (28.1)	0.56
Diabetes mellitus	22 (36.7)	16 (28.1)	0.43
Insulin dependent	6 (27.3)	3 (18.8)	0.89
Hypertension	55 (91.7)	49 (86.0)	0.49
Hyperlipidemia	51 (85.0)	45 (78.9)	0.54
Smoking	27 (45.0)	26 (45.6)	0.63
Family history of CAD	21 (35.0)	20 (35.1)	1.00
Prior myocardial infarction	9 (15.0)	12 (21.1)	0.40
Prior bypass surgery	3 (5.0)	3 (5.3)	1.00
Prior percutaneous coronary intervention	30 (50.0)	28 (49.1)	0.84
Number of vessels diseased			0.72
1 vessel disease	15 (25.0)	18 (31.6)	
2 vessel disease	21 (35.0)	19 (33.3)	
3 vessel disease	24 (40.0)	20 (35.1)	
Clinical presentation			0.61
ST elevation myocardial infarction ≤ 48 h	0 (0.0)	1 (1.8)	
Non ST elevation myocardial infarction	6 (10.0)	4 (7.1)	
Unstable angina	14 (23.3)	11 (19.6)	
Stable angina	21 (35.0)	16 (28.6)	
Asymptomatic	19 (31.7)	24 (42.9)	

Data shown as mean ± SD or number (percentage).

**Table 2:** Baseline culprit lesion and procedural characteristics.

	BP-EES	BRS	*p* Value
Culprit lesions	63	59	
Target vessel			0.43
Left anterior descending	29 (46.0)	25 (42.4)	
Left circumflex	16 (25.4)	11 (18.6)	
Right coronary artery	18 (28.6)	23 (39.0)	
ACC/AHA classification			0.80
A	8 (12.7)	6 (10.2)	
B1	22 (34.9)	26 (44.1)	
B2	29 (46.0)	24 (40.7)	
C	4 (6.4)	3 (5.1)	
Pre-dilation	54 (85.7)	58 (98.3)	0.02
Stent diameter, max (mm)	3.2 ± 0.4	3.2 ± 0.3	0.71
Number of primary devices used			0.34
1 device	51 (81.0)	44 (74.6)	
2 devices	12 (19.0)	13 (22.0)	
3 devices	0	2 (3.4)	
Total stented length (mm)	26.8 ± 10.9	26.7 ± 12.1	0.98
Overlap	15 (23.8)	16 (27.1)	0.76
Post-dilation	41 (65.1)	52 (88.1)	<0.01
Nominal diameter balloon, max (mm)	3.4 ± 0.7	3.5 ± 0.3	0.63
Balloon pressure, max (atm)	16.5 ± 4.8	18.2 ± 4.5	0.10
*Quantitative coronary angiography analysis*			
Pre-intervention			
Reference diameter (mm)	2.92 ± 0.41	2.91 ± 0.43	0.94
Minimal lumen diameter (mm)	1.18 ± 0.34	1.17 ± 0.40	0.81
Diameter stenosis (%)	59.5 ± 12.3	60.3 ± 12.8	0.73
Lesion length (mm)	12.3 ± 5.5	13.1 ± 5.7	0.41
Post-intervention			
Minimal lumen diameter (mm)	2.75 ± 0.36	2.65 ± 0.42	0.15
Diameter stenosis (%) (in-stent)	10.2 ± 4.5	13.7 ± 6.0	<0.001

Data shown as mean ± SD or number (percentage).

**Table 3:** Angiographic follow-up at 6 months.

	BP-EES	BRS	*p* Value
Lesions/patients assessed	44	46	
Days to angiographic follow-up	192 [180, 218]	199 [186, 221]	0.19
*In-segment analysis*			
late lumen loss (mm)	0.15 ± 0.33	0.32 ± 0.52	0.07
minimal lumen diameter (mm)	2.32 ± 0.44	2.18 ± 0.64	0.23
diameter stenosis (%)	23.6 ± 10.2	27.6 ± 16.6	0.17
binary restenosis	1 (2.3)	6 (13.0)	0.12
*In-stent analysis*			
late lumen loss (mm)	0.13 ± 0.26	0.21 ± 0.47	0.31
minimal lumen diameter (mm)	2.64 ± 0.39	2.44 ± 0.65	0.08
diameter stenosis (%)	12.5 ± 7.7	19.3 ± 16.5	0.01
binary restenosis	0 (0.0)	5 (10.9)	0.07

Data shown as mean ± SD or median [IQR] or number (percentage); *p*-values are derived from superiority testing.

**Table 4:** Clinical results at 12 months and 5 years.

	12-Month Follow-Up				5-Year Follow-Up			
	BP-EES	BRS	Hazard Ratio (95% CI)	*p* Value	BP-EES	BRS	Hazard Ratio (95% CI)	*p* Value
Death	1 (1.7)	1 (1.8)	1.04 (0.07–16.69)	0.98	7 (11.7)	7 (12.3)	1.06 (0.37–3.03)	0.91
Cardiac death	0	1 (1.8)	NA	0.99	3 (5.0)	7 (12.3)	2.47 (0.64.9.57)	0.19
Device-oriented outcomes								
definite device thrombosis	0	0	NA	NA	0	0	NA	NA
probable device thrombosis	0	0	NA	NA	0	0	NA	NA
target vessel myocardial infarction	0	0	NA	NA	0	0	NA	NA
target lesion revascularization	3 (5.0)	6 (10.5)	2.13 (0.53–8.51)	0.29	4 (6.7)	8 (14.0)	2.16 (0.65–7.17)	0.21
device-oriented composite endpoint	3 (5.0)	7 (12.3)	2.48 (0.64–9.58)	0.19	7 (11.7)	15 (26.4)	2.38 (0.97–5.84)	0.06
Patient-oriented outcomes								
myocardial infarction	0	1 (1.8)	NA	0.99	0	1 (1.8)	NA	0.99
patient-oriented composite endpoint	6 (10.0)	10 (17.5)	1.79 (0.65–4.93)	0.26	13 (21.7)	17 (29.9)	1.46 (0.71–3.02)	0.30

Data shown as number (percentages are Kaplan–Meier estimates); device-oriented composite endpoint consists of cardiac death, target-vessel myocardial infarction, target lesion revascularization; patient-oriented composite endpoint consists of death, myocardial infarction, any revascularization; NA = not applicable.

## Data Availability

The authors confirm that the data supporting the findings of this study are available upon reasonable request.

## Supplementary Materials

The following supporting information can be downloaded at: https://www.mdpi.com/article/10.3390/jcm13195949/s1, CONSORT diagram showing the flow of participants through each stage of the ISAR-RESORB randomized trial
