# Supplementary figures and images for: A Prospective, Randomized Trial of Bioresorbable Polymer Drug-Eluting Stents versus Fully Bioresorbable Scaffolds in Patients Undergoing Coronary Stenting
Source: J Clin Med. 2024 Oct 7;13(19):5949. doi: 10.3390/jcm13195949 (PMC11478066; doi:10.3390/jcm13195949)

CONSORT 2010 Flow Diagram

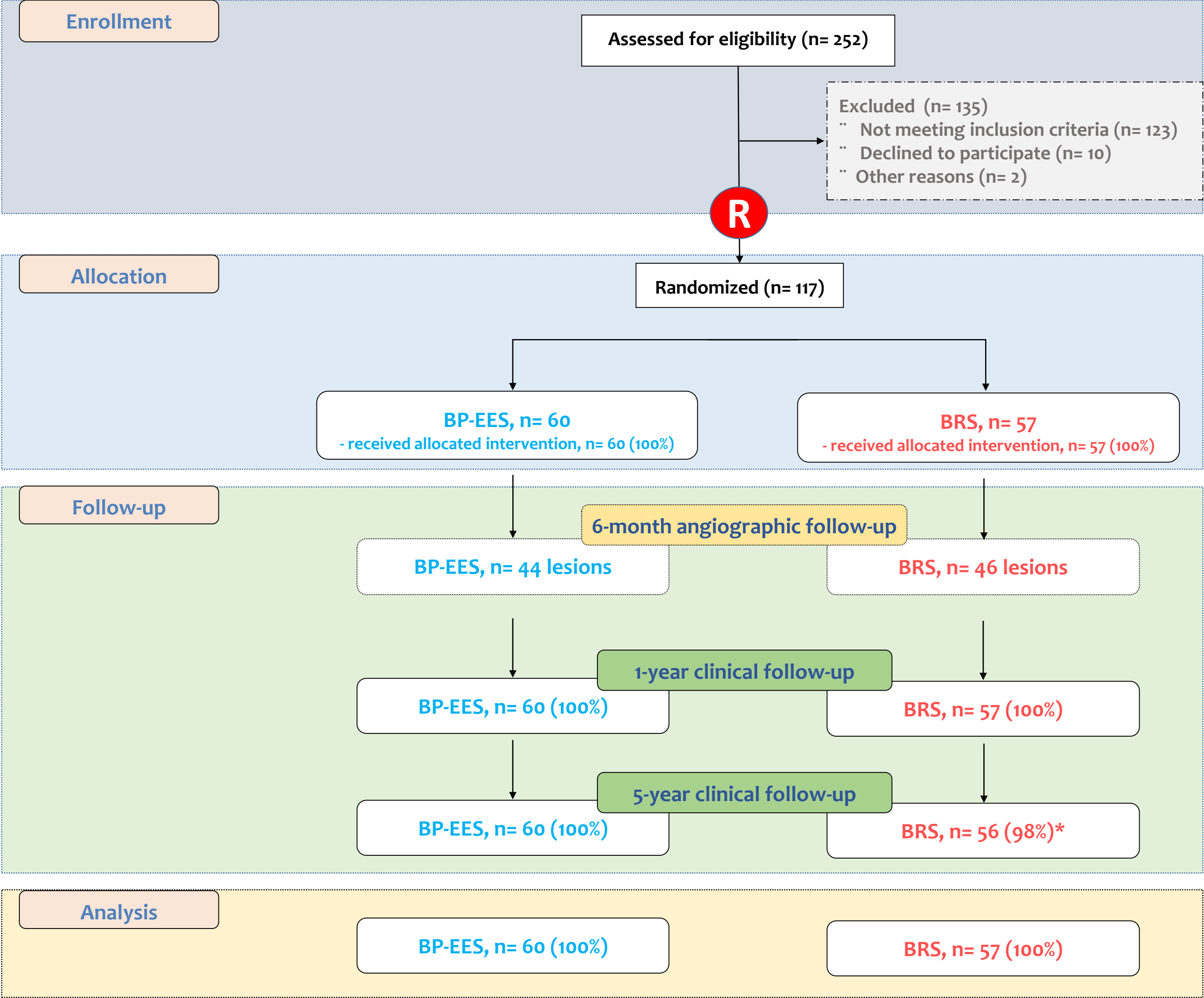

\* 1 patient lost to follow-up due to consent withdrawal

Supplement: Supplementary file 1 [file jcm-13-05949-s001.zip › jcm-3160291-supplementary.pdf]
